# Clinical evolution of neural function in a series of leprosy neuropathy cases after ulnar neurolysis^☆^^☆☆^^★^

**DOI:** 10.1016/j.abd.2020.07.015

**Published:** 2021-06-05

**Authors:** Juliana Barroso-Freitas, Pedro Arthur da Rocha Ribas, Paula Frassinetti Bessa Rebello, Silmara Navarro-Pennini

**Affiliations:** a Hospital Universitário Getúlio Vargas, Manaus, AM, Brazil; b Universidade Nilton Lins, Fundação de Dermatologia Tropical e Venereologia Alfredo da Matta, Fundação de Amparo à Pesquisa do Estado do Amazonas, Manaus, AM, Brazil; c Fundação de Dermatologia Tropical e Venereologia Alfredo da Matta, Manaus, AM, Brazil

Dear Editor,

Leprosy is a chronic infectious disease with a disabling potential due to the affinity of the agent, *Mycobacterium leprae*, for peripheral nerves and skin. Of the 208,619 new cases detected worldwide in 2018, a total of 11,323 cases already had apparent deformities, of which 2,109 (18.6%) were from Brazil, a figure that was second only to India, with 3,666 new cases with deformities.^1^

Neuropathy in leprosy can occur insidiously, more commonly as multiple mononeuropathy, with the simultaneous involvement of two or more peripheral nerves in different regions of the body, with the ulnar and posterior tibial nerves being the most frequently affected. Neural damage can also often occur during acute episodes of immunological reactions against bacillary antigens that may occur during the course of the disease or even after polychemotherapeutic treatment, in the form of neuritis with edema, vascular obstruction, ischemia, and strangulation in the passage through the osteofibrous channels of the limbs, leading to pain and deficit of function, the main cause of physical disabilities and stigma of the disease.^2^, ^3^

The basis for neuritis treatment is prednisone, used to control the inflammatory process and pain, preventing permanent neural damage. Neurolysis is surgery for nerve decompression through an incision in the epineurium and opening of the osteofibrous canal to release the nerve, at the level of the elbow and/or wrist.^3^ It is performed mainly in cases of nerve abscess, neuritis not responsive to clinical treatment for four weeks, recurrent or subentrant episodes of neuritis, chronic neuritis with deficit and pain, and neuritis in patients with comorbidities that contraindicate the use of corticosteroids aiming to decrease the dose and in cases of corticosteroid contraindications.^4^

With the purpose of describing the clinical evolution of neural function and neuritis recurrences of leprosy patients with neuritis submitted to neurolysis, we present a series of 22 cases of ulnar nerve surgery performed in the years 2015 and 2016, at Fundação de Dermatologia Tropical e Venereologia Alfredo da Matta, located in the state of Amazonas, in the city of Manaus, Brazil.

Immediately before the neurolysis and after it (30, 90 days and according to the follow-up), the Simplified Neurological Assessment was carried out according to the protocol of the Ministry of Health of Brazil.^4^ Sensibility evaluation was performed with an esthesiometer or Semmes-Weinstein monofilament, which consists in a set of six nylon threads of different colors and thicknesses that, pressed against the skin, correspond to different weights (from 0.05 g to 300 g). The 0.05 g (green) and 0.2 g (blue) monofilaments were considered as normal sensibility and above that, as decreased sensibility.^5^ For the assessment of muscle strength, the abductor muscle of the fifth finger was assessed and the Medical Research Council scale was used, which ranks strength from zero (no muscle movement) to five (complete movement against gravity with maximum resistance).^4^ Levels four (full movement against gravity with partial resistance) and five were considered normal.^5^

The time between the first episode of neuritis and the performance of neurolysis was, on average, 25.3 months (SD = 63.3; Min. = 1; Max. = 303), with the procedure being performed after up to six months in 11 (50.0%) patients, which is related to the best results by other authors.^5^ For comparison purposes, the last neurological evaluation was considered in the present study, which ranged from three to 168 months, with 12 (54.5%) patients having more than one year of follow-up.

Of 12 cases that already had altered sensibility before surgery, seven (58.3%) showed improvement and, of the six that had altered muscle strength, five (83.3%) maintained the same level, and only one (16.7% ) showed worsening. Fifteen (68.2%) patients did not have any neuritis in the operated nerve; however, three of these patients still had to use prednisone, because they had a leprosy reaction and/or neuritis in other nerves. Two patients had only a single episode of neuritis, which occurred three and four months after surgery. Another five patients (18.2%) had subentrant episodes of neuritis in the operated nerve, for an average of 52.6 months (SD = 63; Min. = 17; Max. = 172) after surgery, without necessarily developing loss of function (Table 1).

**Table 1:** Characteristics of leprosy cases submitted to ulnar neurolysis.

Numerical order	Sex	Age at diagnosis (years)	Clinical form	Time in months between		Sensibility^a^		Muscle strength^b^		Time with recurrences (months)
				Neuritis and neurolysis	Neurolysis and evaluation	Initial	Final	Initial	Final	
1	M	31	BL	01	03	0.05 g	0.05 g	4	5	–
2	M	22	BL	02	20	0	10 g	4	5	21
3	M	34	LL	02	05	0.05 g	0.2 g	5	5	–
4	M	8	TT	02	44	300 g	10 g	0	0	–
5	M	20	BT	02	08	300 g	0	0	0	–
6	M	26	BL	02	18	0.05 g	300 g	5	4	03^c^
7	M	13	BT	03	34	300 g	300 g	4	5	–
8	M	35	LL	04	14	0.2 g	0.2 g	5	5	–
9	M	39	LL	04	30	0	4g	3	3	–
10	M	49	LL	05	24	0.2 g	0.2 g	5	5	–
11	M	51	BL	6	03	300 g	0	2	2	–
12	M	24	LL	09	26	300 g	0.2 g	5	5	–
13	M	24	LL	10	02	0.2 g	0.2 g	5	5	–
14	M	11	BL	12	21	0.05 g	0.05 g	5	5	21
15	M	36	BL	16	17	300 g	10 g	3	3	17
16	M	15	LL	17	04	0.05 g	0.05 g	5	5	–
17	F	57	BT	20	08	300 g	0.2 g	4	4	–
18	M	18	BL	20	168	300 g	0	3	1	172
19	M	17	LL	31	10	0.05 g	0.2 g	5	5	32
20	F	12	BT	36	04	300 g	4g	4	5	04^c^
21	M	19	BL	49	135	0.05 g	0.05 g	5	5	–
22^d^	M	26	LL	303	03	10 g	10 g	2	2	–

a Monofilament: 0.05 g (green) and 0.2 g (blue) = normal sensibility; 2.0 g (violet) = decrease in protective sensibility, with decreased discrimination of shape and temperature; 4.0 g (dark red) = decreased protective sensibility; 10.0 g (orange) = can feel deep pressure and pain; 300.0 g (magenta red) = loss of deep pressure feeling, can feel pain; and 0 = does not feel any monofilament or absence of sensibility to pressure or pain.

b Medical Research Council scale.

c Month of occurrence of the single episode of neuritis.

d This patient had a recurrence.

This series of cases emphasizes the clinical importance of the results in a routine situation, where the greatest contribution of neurolysis associated with the clinical treatment of leprosy neuritis was the non-recurrence or non-chronicity of the condition. Neurolysis also prevents prolonged corticosteroid therapy and its consequences, in addition to the possible evolution into physical disabilities. There was sensory function gain in most of the assessed cases. However, it is necessary to carry out studies with appropriate methodology to evaluate the effectiveness of neurolysis or its benefits in comparison to clinical treatment alone, even considering the studies that have been published for decades.^5^

## Financial support

10.13039/501100004916Fundação de Amparo à Pesquisa do Estado do Amazonas- undergraduate scholarship.

## Authors’ contributions

Juliana Barroso-Freitas: Design and planning of the study; drafting and editing of the manuscript; collection, analysis, and interpretation of data; critical review of the literature.

Pedro Arthur da Rocha Ribas: Critical review of the literature; collection, analysis, and interpretation of data.

Paula Frassinetti Bessa Rebello: Design and planning of the study; critical review of the literature; analysis of data; critical review of the manuscript.

Silmara Navarro-Pennini: Design and planning of the study; drafting and editing of the manuscript; critical review of the literature; analysis of data.

## Conflicts of interest

None declared.
